# Investigating Metabolic Plant Response toward Deoxynivalenol Accumulation in Four Winter Cereals

**DOI:** 10.1021/acs.jafc.3c06111

**Published:** 2024-02-05

**Authors:** Laura Righetti, Francesca Vanara, Renato Bruni, Claudia Sardella, Massimo Blandino, Chiara Dall’Asta

**Affiliations:** † Department of Food and Drug, University of Parma, 43124 Parma, Italy; ‡ Laboratory of Organic Chemistry, Wageningen University, Wageningen 6708 WE, The Netherlands; § Wageningen Food Safety Research, Wageningen University & Research, Wageningen 6700 AE, The Netherlands; ∥ Department of Agricultural, Forest and Food Sciences, University of Torino, Grugliasco 10095, Italy

**Keywords:** plant−pathogen interaction, plant metabolomics, mycotoxins, Fusarium, fungal infection

## Abstract

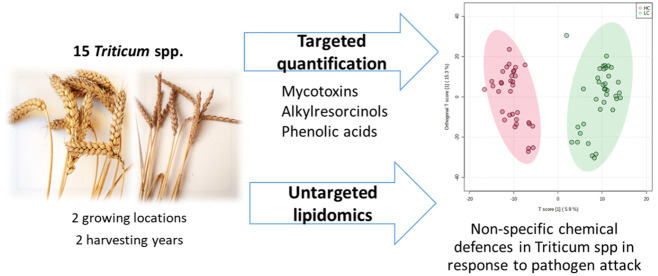

Deoxynivalenol (DON) is a phytotoxic agent supporting the spread of fungal diseases in cereals worldwide, i.e., fusarium head blight. It is known that DON accumulation may elicit changes in plant secondary metabolites in response to pathogen attack. This study maps the changes in selected secondary metabolite classes upon DON contamination occurring in fifteen *Triticum* spp. genotypes, among them emmer, spelt, and soft wheat, and 2 tritordeum varieties, cultivated in two different sites and over two harvest years. The main phenolic classes (i.e., alkylresorcinols, soluble, and cell-wall bound phenolic acids) were targeted analyzed, while changes in the lipidome signature were collected through untargeted HRMS experiments. The results, obtained across multiple *Triticum* species and in open fields, confirmed the modulation of first-line biological pathways already described in previous studies involving single cereal species or a limited germplasm, thus reinforcing the involvement of nonspecific chemical defenses in the plant response to pathogen attack.

## Introduction

Deoxynivalenol (DON) is recognized as the most common mycotoxin in *Triticum* spp. species.^1,2^ It is produced in the field by strains belonging to the genus *Fusarium*, mainly *F. graminearum*, and *F. culmorum*, during each stage of their emibiotrophic life cycle. These pathogens are also responsible for fusarium head blight (FHB) and fusarium root rot (FRR), two severe plant diseases affecting wheat and barley crops worldwide.^3^ Occurrence of DON in edible cereals poses health concern for both human and animals and causes economical losses due to reduced yields and noncompliant food batches.^4^

Several strategies have been proposed to mitigate the impact of *Fusarium* mycotoxins in cereals, and at preharvest stage plant breeding for resistant varieties represents the main pillar.^5^ Plant resistance to FHB is a highly complex quantitative trait controlled by multiple genes, depending on environmental and genotype × environment interactions.^3^ In this respect, biodiversity is considered a valuable source and therefore the genetic pools of minor cultivars, wild relatives, and ancient *Triticum* genotypes are so far actively explored.^6,7^

Together with genomics and transcriptomics studies, metabolomics has recently emerged as a technique of election for diving into such genetic pools and to explore plant adaptation to biotic and abiotic stresses.^8−10^ It is indeed recognized that a large set of constitutive as well as inducible defense metabolites could play a pivotal role in the resistance of cereals against pathogenic fungi.^8,11−15^ Following a comparative approach, many studies have pointed out a wide spectrum of primary and secondary metabolites whose production differs in resistant and susceptible cultivars or that are differently accumulated upon *Fusarium* infection in the field.^14,16,17^

Many reports support for instance the involvement of phenylpropanoids and phenols such as alkylresorcinols (ARs) in plant resistance to fungal pathogens,^18^ which mainly results from their antimicrobial properties, their key role as plant defense mediators and their participation to cell wall reinforcement. Similarly, soluble and cell-wall bound phenolic acids have been described as involved in cell-thickening processes following pathogen insult.^19^

Unsaturated fatty acids also play an important role as constitutive defense metabolites, mainly due to their antimicrobial role toward fungal pathogens,^20^ as modulators of reactive oxygen species (ROS)-production,^21^ and as constituents of the cuticle,^22^ a physical barrier to the pathogen attack. In addition, linoleic (C18:2) and linolenic acid (C18:3) are well-known substrates for lipoxygenases to the formation of oxylipins, fundamental mediators of the lipid signaling cascade in plants.^23^ At the same time, the complex interplay between plants and pathogenic *Fusarium* strains leads to multiple and very dynamic interactions, in which chemical plant defenses intervene lowering the damages mediated by mycotoxins before and after fungal attack, while simultaneously mycotoxins such as DON disrupt such metabolic machinery. The powerful opportunities opened by the availability of metabolomic approaches have induced large expectations on the elucidation of such complex interplay, leading to hypothesizing the possibility to pinpoint specific phytochemical markers of *Fusarium* infection.

The quest for metabolic markers to be used for selection in combination with genetic ones is seemingly facing major difficulties. In particular, a specific scrutiny of the literature evidence a lack of harmonized workflows, protocols, and database, thus weakening the reliability of comparisons between different studies.^24^ In addition, studies are often designed to consider only specific cultivars or species rather than evaluating the consistency of the identified markers across a whole genus. It should also be noticed that resistant-related metabolites are closely dependent on phenotypic plasticity, that is, on the multifactorial plant response to a variety of concurrent and interlaced environmental conditions. The reality is described by scattered and rarely comparable data sets, whose reliability is lowered by multiple uncertainties and by investigations that try to compare different single genotypes grown in different environmental and cultural conditions.

Although a large number of studies have been published over the past decade, none of them compared with a side-by-side approach to the conservation of putative resistant metabolites across a range of *Triticum* species. Therefore, the aim of this study was to identify metabolites commonly and transversely involved in the plant response toward DON accumulation in different winter cereals. At this aim, 15 *Triticum* spp. genotypes, among them emmer (*T. turgidum* spp. *dicoccum*), spelt (*T. aestivum* spp. *spelta*), soft wheat (*T. aestivum* spp. *aestivum*), and 2 tritordeum (× *Tritordeum martinii*) varieties, were cultivated in two different sites and over two harvest years. Several key metabolites including bound and free phenolic acids, phenylpropanoids, and alkylresorcinols were target-analyzed and related to DON contamination. Finally, the metabolomics profile of a representative subgroup was collected and analyzed for the identification of common over-/under-biosynthesized metabolites following DON accumulation. To the authors’ best knowledge, this is the first time that key resistance metabolites were identified across multiple *Triticum* species and in open fields.

## Materials and Methods

### Chemicals and Reagents

5-Nonadecyl-resorcinol, 5-heneicosylresorcinol, 5-tricosyl-resorcinol, 5-heptadecylresorcinol (10 mg powder), 2,5-dihydroxybenzoic acid (DHB), and phenolic acid standards (caffeic acid ≥98%, *p*-coumaric acid ≥98%, ferulic acid ≥99%, gallic acid ≥99%, protocatechuic acid ≥99%, *p*-hydroxybenzoic acid ≥99%, sinapic acid ≥98%, syringic acid ≥95%, and vanillic acid ≥97%) were purchased from Sigma-Aldrich (Steinheim). Analytical standards of DON (100 mg L^–1^ in acetonitrile) and deoxynivalenol-3-glucoside (DON3Glc) (50.6 mg L^–1^ in acetonitrile) were purchased from Romer Laboratories (Tulln, Austria).

LC–MS grade methanol, ethyl acetate, and 2-propanol were purchased from Scharlab Italia Srl (Milan, Italy); bidistilled water was obtained using a Milli-Q system (Millipore, Bedford, MA, USA). MS-grade ammonium formate and formic acid from Fisher Chemical (Thermo Fisher Scientific, Inc., San Jose, CA, USA) were also used.

### Sampling Plan

Fifteen winter varieties of *Triticum* spp. and two of tritordeum were considered for this study (Table 1), and each of them was cultivated side by side in two sites and over two harvest seasons, under natural fungal infection for a total of 204 samples. The field experiment was arranged according to a randomized block design, with three replications of each genotype, referring to soft wheat (*n* = 132), emmer (*n* = 24), spelt (*n* = 24), and tritordeum (*n* = 24). From the major sample set, a subset (*n* = 71) was created for untargeted metabolomics analysis, considering only one geographical area (Cigliano) over two harvest seasons, considering soft wheat (*n* = 42), emmer (*n* = 12), spelt (*n* = 12), and tritordeum (*n* = 12).

**Table 1:** *Triticum* spp. Varieties Included in the Study

species	cultivar	seed company	year of release
Einkorn			
*T. monococcum* spp. *monococcum*	monlis	Prometeo, Urbino, Italy	2006
Emmer			
*T. turgidum* spp. *dicoccum*	luni	SIS, San Lazzaro di Savena, Italy	2002
	giovanni Paolo	Apsovsementi, Voghera, Italy	2008
Spelt			
*T. aestivum* spp. *spelta*	BC Vigor	Bc Institute, Zagreb, Croatia	2012
	rossella	Apsovsementi, Voghera, Italy	2016
Common Wheat			
*T. aestivum* spp. *aestivum*	andriolo	Italian local landrace	from XI*X*° century
	gentilrosso	Italian local landrace	from XI*X*° century
	frassineto	Italian local landrace	1922
	verna	Italian local landrace	1953

	bologna	S.I.S., San Lazzaro di Savena, Italy	2002
	aubusson	Limagrain Italia, Fidenza, Italy	2003
	solehio	Agroalimentare Sud Spa, Melfi, Italy	2008
	arabia	Apsovsementi, Voghera, Italy	2009

	bonavita (yellow grained)	Osivo a. s., Zvolen, Slovakia	2011
	rosso (purple grained)	Saatbau, Leonding, Austria	2011
	skorpion (blue grained)	Agricultural Research Institute, Kromeriz, Czech Republic	2013
Tritordeum			
*x. Tritordeum martinii*	aucan	Agrasys SL, Barcelona, Spain	2011
	bulel	Agrasys SL, Barcelona, Spain	2011

Each selected cultivar was simultaneously cultivated over two growing seasons (2016–2017 and 2017–2018) in two different locations in the Northwest Italian plains, namely, Carmagnola (44°50′ N, 7°40′ E; elevation of 245 m, deep fertile silty-loam soil) and Cigliano (45°18′ N, 8°01′ E; elevation of 237 m, in shallow loam soil), with a lower cation-exchange capacity and organic matter content. Each plot had a 7 × 1.5 m^2^ size. The same agronomic technique was adopted for all cultivars, in particular the winter cereals were sown after soil plowing and the incorporation of maize previous crop debris into the soil, and no fungicide were applied to control foliar or head diseases.

The whole plots were harvested using a Walter Wintersteiger cereal plot combine harvester (Ried im Innkreis, Austria). The grain yield (GY) was calculated on a plot basis and adjusted to a 13% moisture. After harvesting, the husks of emmer and spelt were removed through a laboratory dehusking machine (FC2K Otake, Dellavalle Srl, Mezzomerico, Italy). The thousand-kernel weight (TKW) was determined on two 200 kernel sets of each sample (only whole seeds without husks were considered), using an electronic balance (Scout STX422, Ohaus Europe Gmbh, Nänikon, Switzerland). At least 3 kg of kernels of each plot were milled through a laboratory centrifugal mill (model ZM-200, Retsch, Haan, Germany) equipped with a 1 mm sieve and whole grain flour was carefully homogenized. Prior to chemical analyses, all the samples were further ground to a fine powder (particle size of <250 μm) with a Cyclotec 1093 sample mill (Foss, Padova, Italy) and stored for 2 weeks at −25 °C until the beginning of the analyses.

### Soluble and Cell Wall-Bound Phenolic Acids

Extraction and quantification of the soluble (free and conjugated, SPAs) and cell wall-bound phenolic acids (CWBPAs) were performed as reported in Giordano et al.^25^ The SPAs were determined after alkaline hydrolysis of an ethanol:water extract (80:20, v/v). For the CWBPAs, the alkaline hydrolysis was carried out on the solid sample residue of the ethanol:water (80:20, v/v) extraction. After the acidification of the hydrolysates and liquid–liquid extraction with ethyl acetate, the organic phase was evaporated to dryness under a nitrogen stream. The dry residue was reconstituted with 80:20 (v/v) methanol:water solution, filtered, and analyzed by means of high-performance liquid chromatography with diode array detection (HPLC-DAD).

### Alkylresorcinol Analysis

ARs were extracted following Pedrazzani et al.^26^ and analyzed according to Righetti et al.^27^ Briefly, 1 g of whole grain flour was stirred for 60 min at 240 strokes min^–1^ with 20 mL of ethyl acetate and then centrifuged for 10 min at 14 000 rpm (21 952 *g*). The supernatant (1000 μL) was dried under a nitrogen flow.

After two repetitions, the supernatants were pooled, reconstructed into 1 mL of mobile phase B, and injected into the UHPLC–TWIMS–QTOF. AR quantification was based on external standard calibration (range of 0.1–25 mg kg^–1^), and it was performed based on our previous study.^26^ The ratio between AR 21:0 and 23:0 was then calculated and used as an indicator for the increased antimicrobial capacity of the plants. Previous results *in vitro*(28) reported AR21:0/AR23:0 as an indicator of antifungal activity and to negatively correlated with DON in an open field study.^27^

### Untargeted Lipidomics

The same grain extract of AR underwent lipidomics analysis. An Acquity I-class UPLC separation system coupled to a Vion IMS QTOF mass spectrometer (Waters, Wilmslow, UK) equipped with an electrospray ionization (ESI) interface was employed for AR profiling. Samples were injected (1 μL) and chromatographically separated using a reversed-phase C18 BEH Acquity column (2.1 × 100 mm^2^, 1.7 μm particle size) (Waters, Milford, MA, USA). Gradient elution was set according to Righetti et al.^27^

Mass spectrometry data were collected in negative electrospray mode over the mass range of *m*/*z* 100–1100. Source settings were maintained by using a capillary voltage of 2.5 kV, a source temperature of 120 °C, a desolvation temperature of 500 °C, and a desolvation gas flow of 1000 L h^–1^. A TOF analyzer was operated in sensitivity mode, and data were acquired using HDMSE, which is a data-independent approach (DIA) coupled with ion mobility. The optimized ion mobility settings included a nitrogen flow rate of 90 mL min^–1^ (3.2 mbar), a wave velocity of 650 m s^–1^, and a wave height of 40 V. The TOF was also calibrated prior to data acquisition and covered the mass range from *m*/*z* 151 to 1013. TOF and CCS calibrations were performed for both positive- and negative-ion mode. Data acquisition was conducted using a UNIFI 1.8 (Waters, Wilmslow, UK).

Data processing and compound annotation were conducted using Progenesis QI Informatics (Nonlinear Dynamics, Newcastle, UK) as previously reported by our group.^16^ Briefly, each UHPLC-MS run was imported as an ion-intensity map, including *m*/*z* (*m*/*z* range 100–1100) and retention time, that were then aligned in the retention-time direction (0.5–16 min). PCA with pareto scaling was performed to check the quality of the raw data and afterward, the variables were filtered, retaining entities with coefficients of variation lower than 30% across the QCs. From the analysis of the variance (ANOVA) significant features were selected (Benjamini–Hochberg false discovery rate adjusted *p*-value <0.01). The resulting significant features to both were subjected to the annotation by publicly available database searches including Lipid Metabolites and Pathways Strategy (LIPID MAPS). Based on the Metabolomics Standards Initiative,^29^ metabolites reported in Table 5 were annotated as level II (putatively identified compounds).

### Mycotoxin Analysis

Samples were extracted and analyzed according to Righetti et al.^27^ Briefly, after grinding, 1 g of whole grain sample was extracted with 4 mL of acetonitrile:water (80:20, v/v) mixture acidified with 0.1% of formic acid, evaporated to dryness, and redissolved in water:methanol (80:20, v/v) prior to LC-MS injection.

The UHPLC-MS/MS analysis was performed on a UHPLC Dionex Ultimate 3000 instrument coupled with a triple quadrupole mass spectrometer (TSQ Vantage; Thermo Fisher Scientific Inc., San Jose, CA, USA) equipped with an electrospray source (ESI). For the chromatographic separation, a C18 Kinetex column (Phenomenex, Torrance, CA, USA) with a diameter of 2.10 × 100 mm^2^ and a particle size of 2.6 μm heated to 40 °C was used.

Two μL of sample extract was injected into the system; the flow rate was 0.350 mL min^–1^.

Gradient elution and MS detection was performed as previously described^27^ by using 5 mM ammonium acetate in water (eluent A) and methanol (eluent B), both acidified with 0.2% acetic acid. Detection was performed in SRM mode, operating in negative ionization mode, as previously described. Matrix-matched calibration curves (calibration range 50–1000 μg kg^–1^) were used for target analyte quantification. DON was quantified together with its major masked form, DON-3-glucoside (DON3Glc).

### Statistical Analysis

Statistical analyses were performed using Statistica 13.5.0.17 (Tibco, Palo Alto, CA, USA). Data were analyzed by Full Factorial ANOVA followed by Tukey’s post hoc test as well as for Pearson’s correlation with α = 0.05 in both cases.

The data set was then exported into MetaboAnalyst 4.0,^30^ log-transformed, and Pareto-scaled before evaluating the quality of the unsupervised and supervised models. Principal component analysis (PCA) was performed to assess the natural sample grouping. Significant variables were selected, according to the FDR corrected value of *p* < 0.01.

## Results and Discussion

A total of 204 small cereal grain samples were collected from experimental fields over two harvest seasons (2017 and 2018) and two sites (Carmagnola and Cigliano), located in a geographical area in Northwest Italy. The sample set was analyzed for DON and DON3Glc content as well as for the polar and apolar phenolic compounds profile, to identify any potential correlation between small secondary metabolites involved in plant defense and DON occurrence in the field. In addition, a sample subset (*n* = 71) underwent untargeted HRMS lipidomics analysis to highlight consistent changes in the lipidome profile potentially ascribed to DON accumulation.

### DON and Phenolic Compound Content in *Triticum* Samples

All samples harvested in 2017 and 2018 were found to be contaminated with DON ranging from 20 to 30 780 μg kg^–1^ (median value: 1109.9 μg kg^–1^) as well as with DON3Glc in the range 20–3232 μg kg^–1^ (median value: 94.1 μg kg^–1^). Based on a full factorial ANOVA, DON contamination was dependent on the harvest year (*p* < 0.0001) and on the crops (*p* = 0.0318), while no significant difference was found for the site (*p* = 0.3955) and for interactions among factors. The same trend was observed for DON3Glc. Aggregated results based on the species are reported in Table 2. DON and DON3Glc concentration are positively correlated, as already reported in the literature^31^ (Pearson’s *r* = 0.9161, *p* < 0.001). Noteworthy, while the 2017 harvest campaign was characterized by an overall mild contamination, DON content in 2018 was generally higher, because of higher rainfall in April, which favored higher *Fusarium* inoculum, and above all in May, which caused a higher head infection at flowering (see Supporting Information (SI), Table S1). These results are consistent with previous studies,^32^ showing the strong influence of the meteorological trend of growing season on mycotoxin accumulation in grains. No significant differences are observed between the two geographical areas, according to a similar meteorological trend recorded in both locations, in particular, as far as the rainfall around flowering and early maturity growth stages is concerned.

**Table 2:** Deoxynivalenol Contamination, Content in Bioactive Compounds, Aggregated Grain Yield, and Thousand Kernel Weight Data According to the Species^a^

	N	DON (μg/kg)	DON3Glc (μg/kg)	total ARs (mg/kg dw)	SPAs (mg/kg dw)	CWBPAs (mg/kg dw)	GY (t/ha)	TKW (g)
*T. aestivum*	132	3030.2 ± 450.4b	326.6 ± 44.6b	1099.4 ± 35.6b	87.4 ± 4.1	644.6 ± 12.8a	4.0 ± 0.1a	42.9 ± 0.6b
*T. dicoccum*	24	2251.8 ± 459.8c	217.1 ± 38.9c	969.6 ± 25.0c	95.1 ± 6.3	543.8 ± 31.6b	3.4 ± 0.2b	47.9 ± 1.2a
*T. spelta*	23	2579.7 ± 671.9c	148.7 ± 45.3d	899.2 ± 57.8c	72.2 ± 5.2	642.1 ± 28.3ab	4.4 ± 0.1a	51.1 ± 0.9a
*x Tritordeum martinii*	24	5141.9 ± 1001.0a	404.3 ± 102.3a	1235.6 ± 103.8a	87.8 ± 10.1	627.6 ± 26.5ab	3.0 ± 0.2b	34.0 ± 1.2c

2017		353.5 ± 40.2b	42.1 ± 7.8b	1029.4 ± 47.1	51.6 ± 1.4b	525.0 ± 8.5b	4.4 ± 0.1a	46 ± 0.6a
2018		5892.8 ± 535.7a	560.7 ± 52.9a	1125.1 ± 29.2	121.4 ± 3.5a	734.7 ± 11.7a	3.4 ± 0.1b	40.5 ± 0.8b

Cigliano	102	3038.7 ± 523.7	331.7 ± 54.6	999.2 ± 25.6b	79.7 ± 3.9b	611.7 ± 12.7b	3.9 ± 0.1	47 ± 0.7a
Carmagnola	101	3235.8 ± 410.5	273.3 ± 34.8	1156.5 ± 48.3a	93.7 ± 4.7a	649.3 ± 16.1a	3.9 ± 0.1	39.4 ± 0.7b

a Abbreviations: DON = deoxynivalenol; DON3Glc = deoxynivalenol-3-glucoside; ARs = alkylresorcinols; SPAs = soluble phenolic acids; CWBPAs = cell wall-bound phenolic acids; GY = aggregated grain yield; TKW = thousand kernel weight. Data are expressed as the mean ± SE on a dry weight basis. Different letters indicate significant differences.

**Figure 1:**
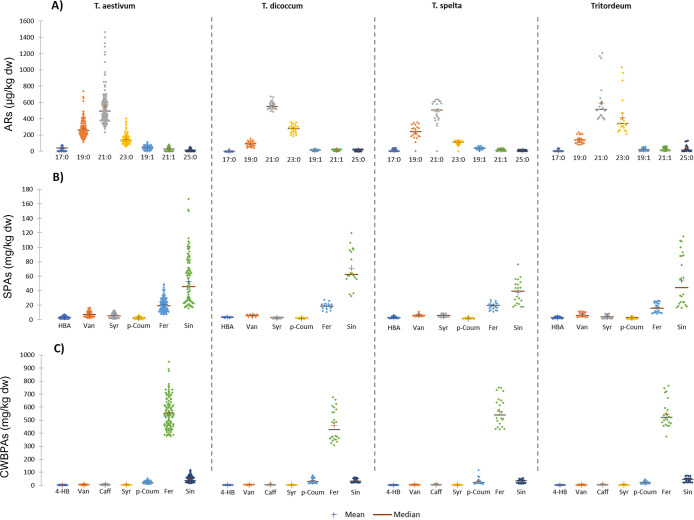
Phenolic compounds content in different *Triticum* species: (A) alkylresorcinols (ARs), (B) soluble phenolic acids (SPAs), and (C) cell wall bound phenolic acids (CWBPAs). Abbreviations: hydroxybenzoic acid = HBA; vanillic acid =Van; caffeic acid = Caff; syringic acid = Syr; p-coumaric = p-Coum; ferulic acid = Fer; sinapic acid = Sin.

Being the most reported metabolites reported in the literature when wheat resistance is considered, ARs and phenolic acids were quantified in the current study. Aggregated data based on crops are reported in Table 2, while an overview of the phenolic composition in each species is reported in Figure 1 as a box plot. The full data set is available as SI, Table S2.

The ARs profile was collected based on the method proposed by Pedrazzani et al.^26^ Based on a full factorial ANOVA, significant differences in total ARs content (as the sum of AR17:0, AR19:0, AR21:0, AR23:0, AR25:0, AR19:1, and AR21:1) according to the site (*p* = 0.0052) and the crop (*p* = 0.0064), the harvest year, and the interactions between factors were found to significantly affect the ARs content. In particular and as previously reported in uninfected plants, the total ARs content is significantly higher in tritordeum compared to other species, while the lowest content has been obtained for both harvest seasons in spelt.^26^ Distribution of major ARs in each species is reported as a box plot in Figure 1A.

As far as the polar fraction, both soluble and bound phenolic acids were analyzed. The full factorial ANOVA returned for the SPAs, expressed as the sum of all the soluble phenolic acids, the site and the year as significant factors (site, *p* = 0.0001; harvest year, *p* < 0.0001) as well as the interaction species*year (*p* = 0.0005), while no significant difference was found among crops. Concerning CWBPAs, expressed as the sum of all the insoluble-bound phenolic acids, all factors as well as the species*year interaction were found to exert a significant effect (site, *p* = 0.0073; harvest year, *p* < 0.0001; species, *p* < 0.0001; species*year, *p* = 0.0462). Differences among species can be observed in Figure 1B,C. Considering that phenylpropanoid compounds are constitutive metabolites in *Triticum* spp. and they can be induced by specific biotic and abiotic elicitation (such as overall microbial exposure, drought and salinity, for instance), our data are consistent with an accumulation based on the genetic background and its interaction with the environment.^17,33^

To investigate potential correlation among polar and apolar phenolic compounds and total DON accumulation (as the sum of DON and DON3Glc) in each species, collected data underwent correlation analysis (Pearson’s test, α = 0.05), as reported in Table 3.

**Table 3:** Pearson’s Correlation Values among Phenolic Compounds and DON Contamination^a^

		*T. aestivum*		*T. dicoccum*		*T. spelta*		tritordeum	
correlation with DON	harvest year	*p*	*r*	*p*	*r*	*p*	*r*	*p*	*r*
total ARs	2017	–0.0666	0.5953	–0.5142	0.0872	–0.3144	0.3463	**0.8736**	**0.0002**
C 21:0/C 23:0		**–0.4029**	**0.0008**	0.1533	0.6344	**–0.8274**	**0.0009**	**–0.7950**	**0.0020**
SPAs		–0.0547	0.6626	–0.4028	0.1942	**0.6581**	**0.0277**	0.3991	0.1987
CWBPAs		0.0241	0.8478	0.3893	0.2110	–0.1066	0.7551	0.3452	0.2718
									
total ARs	2018	0.1012	0.4189	**–0.6839**	**0.0142**	**–0.7609**	**0.0041**	–0.3613	0.2485
C 21:0/C 23:0		–0.1888	0.1290	0.5174	0.0849	0.0238	0.9446	0.0497	0.8781
SPAs		–0.2300	0.0632	–0.5719	0.0521	–0.0010	0.9976	–0.2025	0.5280
CWBPAs		–0.1498	0.2299	–0.2759	0.3853	**–0.6483**	**0.0226**	–0.5317	0.0752

a Significant values are given in bold (α = 0.05).

Overall, a low and not conserved correlation can be observed within crops and across the two harvest years. Although the ARs profile is known to be species-related, the ratio AR21:0/AR23:0 is more conserved among genotypes^26^ and reported as a marker of antifungal activity.^28^ It is noteworthy, based on the collected data, in 2017 the ratio AR21:0/AR23:0 was negatively correlated with DON concentration in 3 out of 4 species considered in this study, while no correlation was observed in 2018.

### Untargeted Lipidomics Analysis

Starting from the initial sample collection, a representative subset (*n* = 71) was selected for untargeted lipidomics analysis, aiming at the identification of secondary metabolites differently accumulated in the two harvest years and based on the DON content. To decrease the variability, only one site (Cigliano) over two harvest seasons was considered, while all the genotypes were maintained (see Table 1 for details). From the main subset, two sample subgroups were selected based on the harvest year, as reported in Table 4.

**Table 4:** Sample Subsets Used for the Untargeted Analysis.

Tag	N	harvest year	species^a^	median [DON] (μg/kg)	mean [DON] (μg/kg)
LC	37	2017	7 TOD; 4 TS; 3 TD; 23 TAE	78 (18–976)	176
HC	34	2018	19 TAE; 6 TOD; 3 TD; 6 TS	5158 (489–28838)	8963

a Tritordeum = TOD; *T. aestivum* spp. *spelta* = TS; *T. turgidum* spp. *dicoccum* = TD; *T. aestivum* spp. *aestivum* = TAE.

Subgroups did not differ in agronomic conditions applied, and the grain yield was fully comparable in Cigliano (on average for all genotypes, 3.8 and 3.9 t/ha in 2017 and 2018, respectively, data not shown). DON concentration was the only relevant factor significantly changing over the two harvest years, being 5158 μg kg^–1^ the median values for subset harvested in 2018 (tag: HC group) and 78 μg kg^–1^ for the subset harvested in 2017 (tag: LC group). This gives rise to subsets with the same genotypes harvested in different years and with very large difference in contamination. Although the authors are aware that the lipidome composition may strongly reflect the species-specific genetic variability, the data mining was set up to explore DON-related differences consistent across the four species.

**Figure 2:**
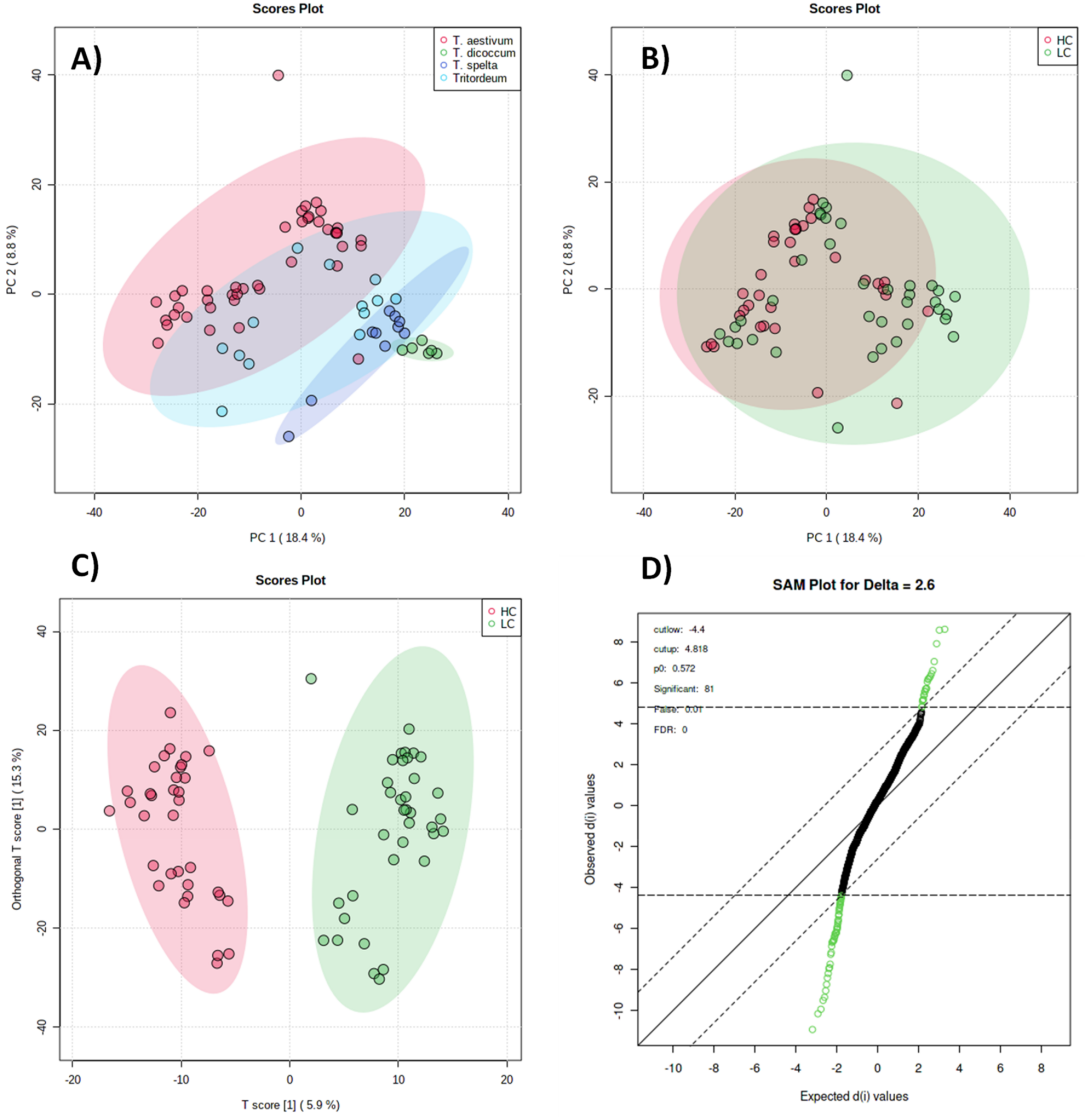
Unsupervised and supervised analyses of selected samples. (A) PCA tagged according to species; (B) PCA tagged according to HC/LC; (C) OPLS-DA based on HC/LC groups; (D) SAM-plot for putative markers selection.

Samples were analyzed by UHPLC-Q-TOF-MS under a fully untargeted approach previously developed by our group.^101−103^ After a quality assessment, data were filtered by choosing entities present with a rate of 100% in at least one sample group, resulting in a reduced data set of 1889 features (the full data set is available as SI, Table S3). The raw data were subjected to PCA to obtain an overview of the trend in an unsupervised manner; as expected, clustering according to the genotype can be appreciated (Figure 2A), while no grouping based on DON accumulation was observed (Figure 2B). However, when a supervised O-PLSDA model was applied (R2X = 0.0592, R2Y = 0.53, Q2 = 0.413), HC and LC groups were efficiently separated, and significant features (*n* = 81) were obtained from SAM-plot (*p* < 0.01). The supervised sample grouping is reported in Figure 2, together with the SAM plot (Figure 2C,D, respectively).

A total of 33 metabolites were annotated as reported in Table 5; the tentative identification was based on the pseudomolecular ion, the retention time, and the composite spectrum of each compound.

**Table 5:** Annotated Metabolites

*m*/*z*	ID	retention time (min)	adducts	formula	mass error (ppm)	anova (*p*)	max fold change
329.2316	stearic acid	2.0	M+FA-H	C18H36O2	–3.8	2.43 × 10^–06^	3.951
311.2221	13-HpODE	2.3	M-H	C18H32O4	–0.6	0.000187	1.653
313.2377	12,13-DiHOME	2.5	M-H	C18H34O4	–2.0	0.002707	1.544
309.2051	13-HpOTrE	2.5	M-H	C18H30O4	–1.9	0.000114	–5.799
233.1527	9-hydroxydecanoic acid	2.7	M+FA-H	C10H20O3	1.3	4.31 × 10^–09^	–2.191
295.2263	13-HODE	2.8	M-H	C18H32O3	–5.1	0.000621	1.610
293.2103	13-OxoODE	2.8	M-H	C18H30O3	–1.9	8.40 × 10^–05^	2.247
315.2523	12,13-dihydroxystearic acid	2.9	M-H	C18H36O4	–1.7	2.53 × 10^–05^	1.925
279.2314	10,13-octadecadienoic acid	4.0	M-H	C18H32O2	–5.5	1.47 × 10^–05^	1.762
277.2167	linolenic acid	4.6	M-H	C18H30O2	–0.6	0.000363	1.753
279.2324	linoleic acid	4.9	M-H	C18H32O2	–0.5	0.002073	1.445
223.0268	esculetin	5.9	M+FA-H	C9H6O4	2.1	0.008928	–2.322
573.4495	DG (15:1/18:3)	6.1	M-H	C36H62O5	–5.0	<1 × 10^–9^	11.550
575.4667	DG (15:1/18:2)	6.3	M-H	C36H64O5	–2.4	<1 × 10^–9^	6.482
551.4649	DG (15:1/16:0)	6.6	M-H	C34H64O5	–5.8	<1 × 10^–9^	13.011
577.4811	DG (15:1/18:1)	6.7	M-H	C36H66O5	–4.5	<1 × 10^–9^	10.836
557.4552	DG (15:0/18:3)	7.3	M-H2O-H	C36H64O5	–3.9	3.00 × 10^–09^	–6.304
600.5180	Cer(t18:0/16:0)	7.4	M+FA-H	C34H69NO4	–5.0	<1 × 10^–9^	4.120
557.4557	DG(15:0/18:3)	7.5	M-H2O-H	C36H64O5	–3.1	4.62 × 10^–08^	2.711
473.3977	sasanquol	7.6	M+FA-H	C30H52O	–5.2	6.90 × 10^–09^	1.353
559.4709	DG(15:0/18:2)	7.6	M-H2O-H	C36H66O5	–3.9	1.56 × 10^–09^	2.178
830.5878	dioleoylphosphatidylcholine	7.9	M+FA-H	C44H84NO8P	–4.9	2.89 × 10^–09^	1.288
535.4695	DG (15:0/16:0)	7.9	M-H2O-H	C34H66O5	–6.5	2.52 × 10^–09^	5.833
989.6390	PI(20:1/22:2)	7.9	M+FA-H	C51H93O13P	5.5	6.94 × 10^–05^	–1.375
561.4861	DG (15:0/18:1)	7.9	M-H2O-H	C36H68O5	–4.6	5.50 × 10^–10^	3.797
991.6486	PI(20:1/22:1)	8.3	M+FA-H	C51H95O13P	–0.5	1.11 × 10^–04^	–1.934
818.6339	GlcCer(d18:0/20:0(2OH))	8.4	M+FA-H	C44H87NO9	2.4	2.34 × 10^–04^	–1.885
682.6329	Cer(t18:0/24:0(2OH))	9.1	M-H,	C42H85NO5	–2.5	9.54 × 10^–04^	1.538
712.6418	Cer(t18:0/24:0)	9.3	M+FA-H	C42H85NO4	–6.4	7.32 × 10^–05^	1.608
869.6488	SM(d18:1/26:1)	9.4	M+FA-H	C49H97N2O6P	–3.3	4.56 × 10^–05^	1.369
857.6491	PA(22:0/22:2)	9.5	M+FA-H	C47H89O8P	2.1	1.51 × 10^–05^	–2.345
870.7524	GlcCer(t18:1(8*Z*)/26:0(2OH))	9.9	M-H–	C50H97NO10	4.8	3.14 × 10^–05^	–5.982

**Figure 3:**
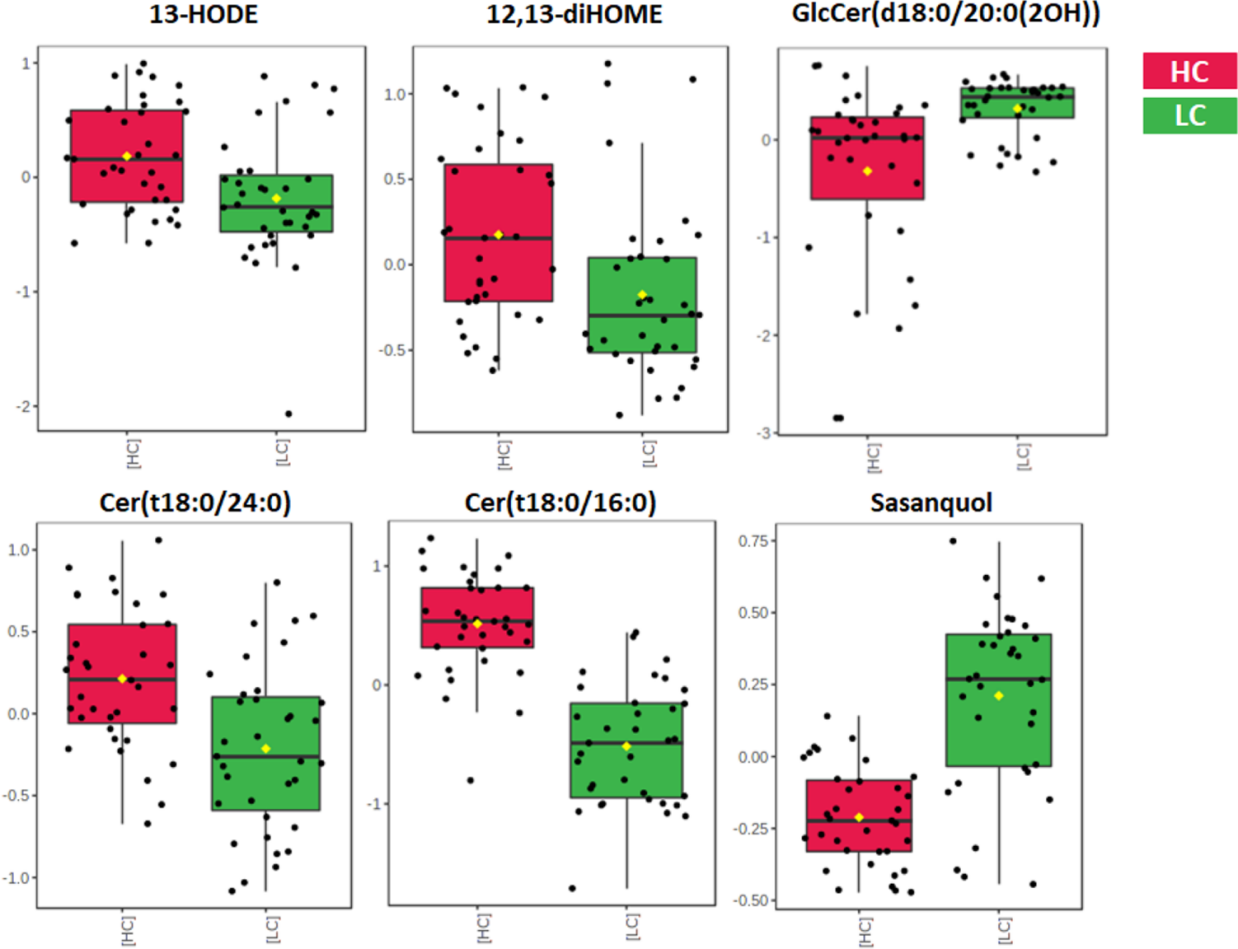
Accumulation of key putative markers in HC and LC groups (normalized data).

Accumulation of selected markers in HC and LC groups is reported in Figure 3. The involved chemical classes are consistent with previous reports, pinpointing once again the central role played by polyunsaturated fatty acids (PUFAs), oxylipins, ceramides, diacylglycerides (DG), and glycerolipids.^16^

The biosynthetic cascade originating from PUFAs is well-known as the front line mechanism counteracting biotic stressors, among them pathogens. During the infection process, initial plant defenses are activated by the detection of ROS that promote lipid peroxidation. As membrane lipids, PUFAs may be released in response to a pathogen attack, triggering the formation of oxygenated FAs and oxylipins, which act as key signaling compounds.^34^ The overaccumulation of C18:2 and C18:3 in the HC group is consistent with the oxylipins signature, these fatty acids being the precursor substrates for oxylipins production. In our study, five oxylipins and two oxidated FAs related to 13-LOX pathway were up regulated for HC group, while two 9-LOX related products are mainly found in LC samples (Table 4). The activation of 13-LOX pathway is also consistent with the accumulation of DGs in HC samples, which can be caused by the alteration of the plant membrane following pathogen attack, as reported by several authors.^23,34−37^

Sphingolipids are also differently accumulated in the two groups. This has been already associated with the accumulation of fumonisins in maize infected by *F. verticillioides*,^38^ as a consequence of the FB-dependent inhibition of ceramide synthase. Because DON does not act as CerS inhibitor, the accumulation of sphingolipids may be linked to programmed cell death mediated by reactive oxygen intermediates, as already reported in *A. thaliana*.^39,40^ In particular, ceramides are higher in HC, while glycosphingolipids are mainly accumulated in the LC group. Again, this is in agreement with an impairment of the sphingolipid pathway following fungal attack, with an hydrolysis of complex sphingolipids and an accumulation of ceramides in the HC group.

**Figure 4:**
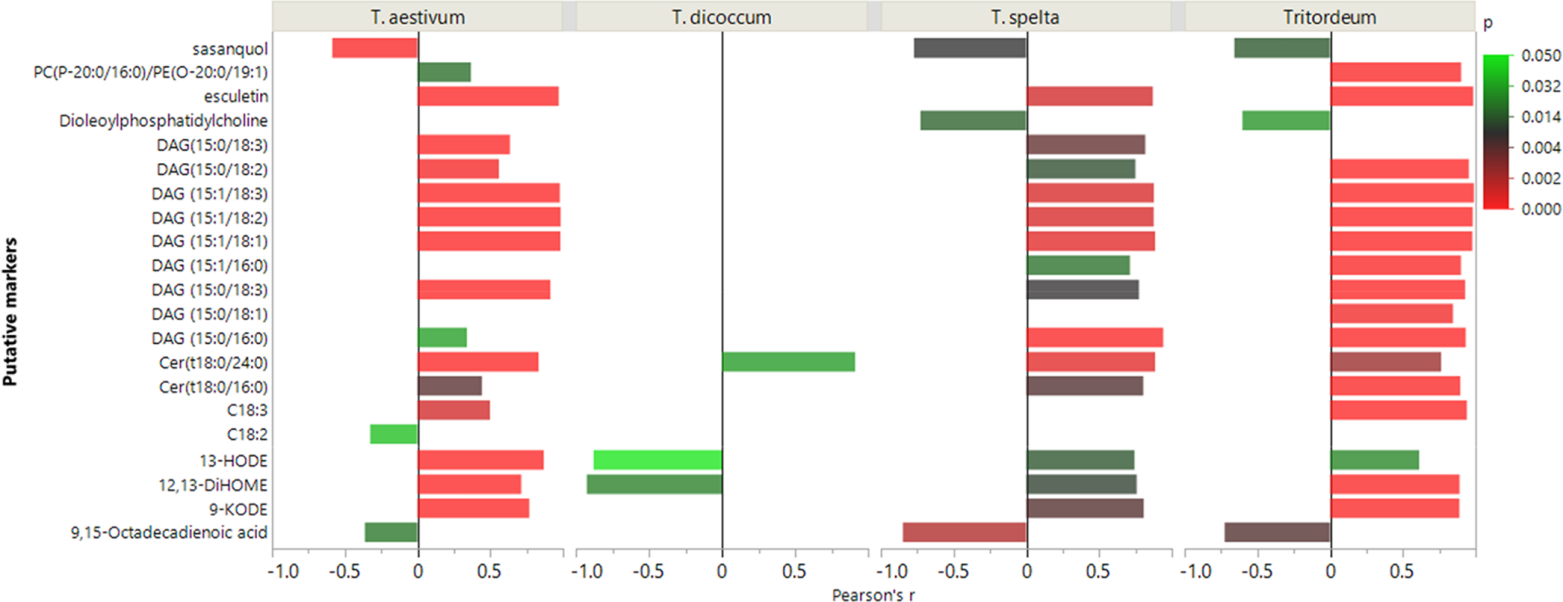
Pearson’s correlation among DON and putative markers in *Triticum* species. Bars represents Pearson’s *r*, while color scale returns *p* values.

Putative markers were then inspected for their correlation with DON accumulation across different crops, as reported in Figure 4. Correlations are generally conserved across hexaploid species, while emmer clearly differs as already observed in the case of phenolic compounds.

### Overall Discussion, Study Limitations, and Significance of the Results

Many papers have attempted to define a set of markers of *Fusarium* infection in wheat, following DON accumulation. However, data reported in the literature are often inconsistent, with compounds showing opposite trends under similar conditions or compounds returning the same trend under apparently different conditions.

This can be explained taking into consideration that in cereals, chemical defense against *Fusarium* and other fungal pathogens is activated by a variety of mechanisms of resistance, acting both pre-emptively and after fungal attack, while operating at anatomical, morphological, physiological, and phytochemical level. These defensive tactics act at different levels and with different timings, and each step may be more or less enhanced and more or less effective according to the stage and the degree of a given pathogen attack.^41^ Secondary metabolites, in particular, are involved in multiple protective functions in response to both biotic and abiotic stress conditions, and it must be pointed out that some of the pathways activated by *Fusarium* and DON exposure may be coelicited by further, different biotic and abiotic stressors such as the overall plant microbiota, drought, or phytophagous insect pressure. The overlap between the effects of drought, salinity, heavy metals, UV-irradiation, herbivory, and exposure to chemicals or other pathogens, may lead to a dynamic in which a single cause–effect relationship may be very difficult to unravel.^42^

Such complexity is particularly hard to capture in open field studies, even with the integration of omics strategies, due to the confounding effect of different layers and timing of (a)biotic stressors the plant is exposed to. While omics workflows are designed to identify effects due to on/off factor (i.e., the presence/absence of a certain stressor or treatment), mycotoxin accumulation following fungal attack should be regarded as a continuous process with a wide spectrum of values modulated by uncontrolled parameters. Even under *in vitro* experiments, the same fungal inoculation may originate different mycotoxins accumulation on plant replicates. Therefore, under natural conditions, where biological replicates are exposed to (slightly) different environmental parameters, both the extent of mycotoxin accumulation and the metabolic fingerprint of the infected plants may present large and sometimes unrelated variations, even on biological replicates.

In addition, because mycotoxin accumulation is strongly affected by meteorological conditions, fungal load, and contamination levels in natural field experiments are often dependent on the harvest season, thus making difficult to isolate the contribution of the pathogen infections to the plant secondary metabolite modulation from the contribution due to other climate-related factors themselves.

The weak consistency in *Fusarium*-infection biomarkers across the literature can be therefore explained in the light of different genotypes, different environments, different (a)biotic stressors, and different fungal loads to which plants are exposed to. It should be noticed that most of the current studies do not consider the overall level of (a)biotic stress the plant undergoes in the field, and therefore it is very difficult to distinguish the plant reaction to the pathogen from its overall, combined reaction to the environment itself. On the other hands, issues with the translation of mechanistic studies using single mycotoxin exposure in plants grown under controlled conditions may hardly offer a real scenario of *Fusarium* infection, in which multiple mycotoxins act in synergy against plant defenses.

Our work, although still suffering from limitations due to uncontrolled open field conditions, has the strength of considering a large sample set consisting of four winter cereal species over 2 years and 2 sites. It confirmed the modulation of first-line biological pathways already described in previous studies involving single cereal species or a limited germplasm, thus reinforcing the involvement of nonspecific chemical defenses.

Although the accumulation of DON is related to an increased exposure of plant cells to ROS, there is no direct cause–effect on the modulation of phenolic compounds biosynthesis. Collected data suggested that the modulation of the phenolic profile should be regarded as an aspecific phenomenon. This would lead to hypothesize its dependence on the overall pressure exerted by pathogens or other stress agents present in the field, that is that a higher or lower abundance of phenylpropanoids and alkylresorcinols may be related to the intensity of the infection rather than on the sole exposure to *Fusarium*.

As far as the lipidomic profile, different compounds were selectively overproduced in plants over the two harvest seasons and showing different DON contents. Their detection was the result of differentials over the two years and thus is representative of distinct external pressure. It is noticeable how the entire lipid defense machinery is activated during the year in which DON is higher in infected plants, confirming a quick action of the defensive frontline which, however, remains nonspecific, being these compounds involved also in the interplay with different pathogens.

Although no drought or thermal stresses were observed over the two years, as indicated by the comparable yield levels between years in Cigliano, we cannot exclude the presence of further biotic and abiotic stress factors that act as confounders inducing a nonspecific and unrelated response in our samples. For instance, water stress is known for inducing an increase in phenylpropanoids and secondary metabolites biosynthesis in general.^43^ In our opinion, this point is often underestimated and, therefore, needs to be explored because it could shed light on inconsistencies emerging in the literature. By neglecting the overall degree of biotic and abiotic stress, it might be very difficult to focus on true markers of *Fusarium* resistance due to confounding factors emerging from co-occurrent stresses.

## Supporting Information Available

Additional experimental details, materials, and methods (PDF)
Table S2 (XLSX)
Table S3 (XLSX)
