# Supplementary material for: Investigating Metabolic Plant Response toward Deoxynivalenol Accumulation in Four Winter Cereals
Source: J Agric Food Chem. 2024 Feb 5;72(6):3200–9. doi: 10.1021/acs.jafc.3c06111 (PMC10870777; doi:10.1021/acs.jafc.3c06111)
Supplement: Supplementary file 2 — jf3c06111_si_002.pdf [file jf3c06111_si_002.pdf]

# Supporting Information

**Investigating metabolic plant response towards deoxynivalenol accumulation in four winter cereals.**

Laura Righetti[1,2,3*], Francesca Vanara[4], Renato Bruni[1], Claudia Sardella[4], Massimo Blandino[4], Chiara Dall'Asta[1*]

[1] Department of Food and Drug, University of Parma, 43124 Parma, Italy

[2] Laboratory of Organic Chemistry, Wageningen University, Wageningen 6708 WE, the Netherlands;

[3] Wageningen Food Safety Research, Wageningen University & Research, Wageningen 6700 AE, the Netherlands

[4] Department of Agricultural, Forest and Food Sciences, University of Torino, Grugliasco 10095, Italy

**Table S1.**

Monthly cumulative rainfall and growing degree days (GDDs)[a] from sowing (November) to the end of the ripening stage (June) measured in the experimental areas.

| Year | Month | Rainfall (mm) | | GDDs (Σ °C·d$^{-1}$) | |
|---|---|---|---|---|---|
| | | Cigliano | Carmagnola | Cigliano | Carmagnola |
| 2016-17 | November | 158 | 257 | 238 | 250 |
| | December | 45 | 77 | 144 | 159 |
| | January | 4 | 12 | 97 | 111 |
| | February | 45 | 62 | 152 | 175 |
| | March | 69 | 69 | 349 | 356 |
| | April | 34 | 51 | 415 | 412 |
| | May | 79 | 77 | 554 | 558 |
| | June | 149 | 103 | 673 | 698 |
| | November - June | 582 | 708 | 2622 | 2718 |
| | April - May | 114 | 128 | 970 | 970 |
| 2017-18 | November | 48 | 66 | 224 | 220 |
| | December | 33 | 27 | 113 | 82 |
| | January | 107 | 117 | 178 | 141 |
| | February | 60 | 86 | 113 | 89 |
| | March | 109 | 103 | 223 | 209 |
| | April | 93 | 116 | 456 | 444 |
| | May | 138 | 310 | 583 | 565 |
| | June | 35 | 14 | 665 | 672 |
| | November - June | 622 | 837 | 2554 | 2421 |
| | April - May | 230 | 425 | 1039 | 1009 |

[a]Accumulated growing degree days for each month using a 0 °C base. Data obtained from the Regione Piemonte agrometeorological service.

**Table S2.** Overview of the phenolic composition in each sample species.

**Table S3.** Untargeted lipidomics dataset of 1,889 features.
